# The rise in narghile (shisha, hookah) waterpipe tobacco smoking: A qualitative study of perceptions of smokers and non smokers

**DOI:** 10.1186/1471-2458-11-315

**Published:** 2011-05-14

**Authors:** Rima T Nakkash, Joanna Khalil, Rema A Afifi

**Affiliations:** 1 Department of Health Promotion and Community Health, Faculty of Health Sciences, American University of Beirut, Beirut, Lebanon

**Keywords:** Waterpipe tobacco smoking, Lebanon, Qualitative Research, FCTC

## Abstract

**Background:**

The prevalence of waterpipe tobacco smoking (WTS) in the Middle East region and worldwide is increasing. There is evidence to indicate both short term and long term health effects of WTS, resulting in the issuance of an advisory note by the World Health Organization.

**Methods:**

This research aimed at gaining an in-depth understanding of the factors contributing to the rise in WTS in Lebanon. Qualitative focus groups (25) and in-depth interviews (9) were conducted with adults in Lebanon in 2007. Participants were recruited to represent diversity in smoking status, gender, age groups and urban/rural residence. The interviews and focus groups were thematically analyzed, and recurrent themes noted and summarized.

**Results:**

The main themes identified were availability, affordability, innovation, influence of media, lack of a policy framework, and the sensory characteristics evoked from WTS. Men and women, smokers and non-smokers, and younger and older participants differed in their emphases on the above themes. These themes, though specific to waterpipe, are similar to themes manipulated by the cigarette industry, and eventually controlled through tobacco control policies.

**Conclusions:**

Understanding reasons behind the rise in waterpipe tobacco use is important if appropriate prevention, cessation, and policy interventions are to be formulated. Strict adherence to the FCTC is warranted, with careful and vigilant attention that all tobacco products are covered by laws in both high as well as middle to lower income countries.

## Background

The prevalence of waterpipe (aka narghile, shisha, hookah) tobacco smoking (WTS) in the Middle East region and worldwide is increasing [1]. Youth WTS is on the rise as compared to cigarette smoking [2,3]. In Lebanon, the Global Youth Tobacco Survey (GYTS), administered to 13-15 year olds in 2005, indicated that 59.8% smoked other forms of tobacco (most likely waterpipe) at least once in the past month as opposed to 10% cigarettes. Compared to 2001, cigarette use among 13-15 year olds in Lebanon has decreased while use of other tobacco products has increased [2]. A cross sectional study of 1964 university students, conducted in Beirut, Lebanon, in 2001, reported use of waterpipe exclusively to be 21.1%, 7.6% only cigarettes, and 11.3% both [3]. In other countries in the region, similar trends are observed. GYTS results evaluating the trends of tobacco use among 13-15 year olds between 1999 and 2008 indicated that in all countries of the Arab region, use of tobacco products other than cigarettes (most likely waterpipe) was more common than use of cigarettes [4]. Although not as widespread in the West, WTS remains an impending threat. In a study in Arizona, USA of 6594 middle and high school students during the 2004-2005 school year, WTS was the third most common source of tobacco consumption after cigarettes and cigars [5]. Similarly in the UK, in a cross-sectional survey of students of Birmingham University, WTS was recognized as an impending public health threat as 37.9% of students had ever tried waterpipe and 8.0% were regular smokers, as compared to 9.4% regular cigarette smokers [6].

WTS is not a safe alternative to cigarettes [7]. Studies using a smoking machine to test toxicant yields in lab environments found that waterpipe tobacco smoke contains carbon monoxide, polyhydrocarbons, formaldehyde, nitrogen, nitric acid, nicotine [7,8] and other toxicants such as arsenic, chromium, lead and volatile aldhehydes [9]. Health risks include [10,11] in the short term developing dependence and acute respiratory diseases and lung impairment. Other more serious negative health outcomes include risk of developing cancers, including lung cancer and other chronic diseases such as cardiovascular and respiratory diseases. During pregnancy, waterpipe smoking can lead to low fetal birth weight [12]. In addition, exposure to second hand smoke from waterpipe smoke poses a serious health risk to non smokers. Other health risks include the spread of infectious diseases, such as Tuberculosis, due to the sharing of the waterpipe among smokers [11]. Despite this knowledge, the health risks of WTS remain largely unrecognized by the lay public [1,13]. For example, a qualitative study in Syria reported people's views of waterpipe smoking as a pleasurable activity among friends with no regard to health consequences [14].

Understanding the factors contributing to the rise in WTS is important to develop properly tailored prevention, cessation, as well as policy interventions. As part of a regional study in Lebanon, Syria, Palestine and Egypt, a qualitative investigation was carried out to understand waterpipe smokers and non smokers' views of the reasons behind the spread of WTS. This manuscript will review the reasons given to explain this increase in WTS in Lebanon.

## Methods

The re-emergence of WTS in the EMR region has been documented [10]. The reasons for this re-emergence and the meaning attributed to WTS have yet to be clearly described. Qualitative methods are uniquely suited to studying phenomena in their naturally occurring setting, and understanding phenomena "in terms of the meanings people bring to them" [15,16]. Focus group discussions (FGD) and in-depth interviews were conducted. Men and women, waterpipe smokers and non-smokers were recruited and assigned to different FGDs by gender and waterpipe smoking status, with conscious intent to include women smokers and non-smokers, and men smokers and non-smokers of waterpipe. As for age, participants from four different age groups were recruited: 18-25 yrs, 26-35 yrs, 36-65 yrs, and 65+ years old. Participants were recruited from 2 different areas in Lebanon: Beirut and Bekaa. Participants from Beirut, living in the capital, were recruited from urban areas; and participants from the Bekaa were mainly recruited from rural areas. Differences in socio-economic status and religion, as well as variability in marital and employment status were monitored throughout the recruitment process. Thus, if we noted that we were getting too many single participants, we would consciously work on approaching married persons to participate. Similarly, if we noted that many participants were employed, we would then focus on recruiting non-employed participants.

Interviews and FGD complemented each other. During interviews, participants tended to share insights related to their personal WTS experience; their connection with the apparatus, their unique reasons for smoking it; while in FGDs, especially when conducted in natural settings - such as in cafés, restaurant, at the university, or at home, the researcher was able to observe the social and cultural dynamics of WTS such as waterpipe smokers' interaction with each other, and with their surrounding environment. Interviews were mainly conducted with persons who were identified to us as heavy waterpipe smokers for a deeper understanding of their experiences.

Participants were recruited in cafés, universities, through home visits, and other public places, following a snowballing technique, where one person would put us in contact with his/her friends who either favoured or disfavoured WTS. Interviews and FGDs were conducted by a researcher holding a Master of Public Health degree and experienced in qualitative method of research. Data collection was stopped when saturation was reached, i.e. no new themes were being generated during the discussions.

A total of 25 FGDs, with 8 to 10 people in each group, and 9 in-depth interviews were conducted in Lebanon, during the summer (July) and the month of Ramadan (mid September - mid October) in 2007. A total of 220 people took part in the interviews and FGDs, with ages ranging between 18 and 74 years old. Few elderly were recruited as they very hard to identify, especially in public places. The majority of the discussions (12) were conducted with participants aged between 26 and 35 years old, followed by the age group 36-65 years old, then 18-25 years old. Twenty interviews and FGD were conducted in the Beirut area and 14 in the Bekaa area. Twenty-one discussions were conducted with waterpipe smokers as opposed to 13 discussions with non-smokers. The same number of discussions (17) was conducted with women and men, separately. Participants from different educational levels and economic statuses were recruited. As for the occupation, students, housewives, employed, unemployed and retired people were all part of the sample. None of the participants who were approached for an interview or to take part in a FGD refused.

The interview guide was pretested prior to data collection and covered the following topics: reasons behind WTS, a description of the state of mind of waterpipe smokers, perceived image of women smoking waterpipe and women smoking cigarettes, as well as the enabling and deterring factors behind WTS. Consent was obtained prior to the initiation of data collection. The researcher would read the consent form to all the participants, explaining the purposes of the research, that participation is voluntary, that they may stop the interview at anytime and that confidentiality of records is ensured. Following their approval to join the discussion, their consent to record the discussion was also obtained. Whenever possible, the interviews and focus groups were conducted in natural settings and in Colloquial Arabic. The discussions were recorded and on average took one hour. All interviews and focus groups were transcribed in their original language in order not to lose meaning in translation. Ethical approval was granted by the American University of Beirut Institutional Review Board.

Thematic analysis was the method of choice to examine the transcripts. It is a process of coding qualitative data using a list of themes or patterns found in the transcripts, ranging from a simple description of observation to interpretation of phenomenon [17]. A coding scheme was developed following a joint meeting of researchers from all countries after deep immersion and multiple readings of transcripts. This allowed for joint practice in coding and subsequent discussions about the appropriateness of each code. Development of codes and themes was inductive and arose from the discussions. The final coding scheme consisted of 11 themes (and 61 subthemes) (Table 1). All the transcripts were then coded by one researcher in Lebanon. Coding is an essential step in the organization, processing and analysis of qualitative information and leads the way for the interpretation phase [17]. Following the analysis of each theme, the research team in Lebanon used to meet and review all the sub-themes and related quotes; disagreements were resolved by discussion among the team members or by going back to the original transcripts and consulting with the field researcher.

**Table 1:** List of themes

Themes that emerged from the regional study	Themes covered in this paper
1. Socio-cultural norms	
2. Sensory Characteristics of waterpipe	✓
3. Pros and cons of waterpipe preparation	
4. Metaphors	
5. Consumerism (Availability, Affordability, Frequency, Cost, Innovation)	✓
6. Indicators of dependence of waterpipe smoking	
7. Comparison between cigarettes and waterpipe	
8. Health effect of smoking	
9. Motivation to smoke (Coping, Entertainment, State of mind, Boredom)	
10. Intervention (Media, Awareness, Policy, Regulations, Government role)	✓
11. Tradition	

This manuscript includes the themes related to sensory characteristics, consumerism, and intervention. To further illustrate themes, we include quotes from the interviews and FGDs. Use of quotes is common in qualitative research to "support researchers claims, .. illuminate experience, evoke emotions .." [18]. Each quote is followed by the gender of the participant, smoking status and age. The quotes that clearly express the themes were selected with variability in the choice of smoking status, gender, and age groups to show discord or unanimity.

## Results

Participants identified the themes of availability, affordability, innovations in design, sensory qualities of WTS smoking, media influence, and policy.

### Availability of the waterpipe in the public sphere

Availability of the waterpipe in the public sphere, particularly in cafés and restaurants was thought to have contributed to the increase in its use and to moving the location of smoking from homes to the public sphere. FGD participants stated that availability contributed towards motivating "those who don't even want to try, to go ahead and try" (female non-smoker - urban area). More women, younger age groups, and smokers noted widespread availability as the reason behind the increase in WTS. An expansion in the availability of waterpipes in food serving establishments was noted across all categories and was described as a recent "trend".

*"...an emerging fad... 10 years ago, we were not used to seeing waterpipes. Nowadays... the first thing you [people] ask about [when entering a restaurant]: do you have waterpipes? ... The waterpipe is fundamental for everything. The restaurant is doing well because it has waterpipes. The evening is going well because the waterpipe is available... in every place there is the waterpipe, in every setting..." *(Female smoker-urban area)

Moreover, FGD participants stated that restaurants and cafés have come to realize that offering waterpipes was a flourishing business. Consequently in response to the demand by customers and competition from others, the business of serving the waterpipe became a lucrative one.

*"What played a big role in making it a "BOOM" is its commercialization. So maybe it began in a certain occasion where they found that they would benefit from it, so they promoted it pretty well...Our restaurants started to serve waterpipes, while earlier we used to go there to have a beer, there were no waterpipes, but afterwards it was on demand, so they promoted it" *(Male smoker - rural area)

Home delivery services have made it accessible at home for people who had otherwise not considered it due to hassles in preparation. WTS requires the purchase of the waterpipe apparatus, the tobacco and all its accessories. It is time consuming and needs a basic level of know-how for preparation - all resolved by the amenities of home delivery. Men more than women, the younger age groups and smokers stated that the advent of waterpipe home delivery services contributed to the increase in use.

### Affordability of waterpipe tobacco smoking

The waterpipe also evolved as an affordable way to spend time in cafés or at home, with little incurred cost. Affordability was brought up more by women and the older age categories. Both smokers and non smokers cited affordability almost equally as contributing to the surge in use. The waterpipe is generally offered in a range of prices starting from L.L. 5,000 and reaching L.L.15,000 ($1 = L.L. 1500) depending on the setting (how fancy and in what neighbourhood). Also, the practice of sharing of the waterpipe meant that smokers could share cost. The duration of a WTS session varied, depending on how often the tobacco was replenished, how many smokers were sharing, and how often they puffed.

*"...we can order it twice, three times...Sharing and if it's for 10,000L.L. and we are 3 or 4 [people], so approximately 2000L.L. per person is affordable" *(Female non smoker - urban area)

For those who smoked at home, it was affordable since, as stated by participants, apart from the tobacco, the accessories can be used repeatedly. Home delivery services were also affordable (L.L. 8,000) [19]. This affordability at home and in public places was also thought to "*encourage young people" *to smoke more (Male smoker - rural area).

### Innovations in designs of the waterpipe apparatus and tobacco flavours

Continuous innovations in the design of the waterpipe apparatus increased marketing potential. The waterpipe apparatus could thus be dressed up or down to suit the taste of the smoker and the context. A café for example transformed the traditional looking waterpipe into a modern aluminium design [20], most likely attractive to trend seeking smokers. Women, the younger age groups, and smokers stated that innovative designs of the apparatus including diverse sizes, colours, and materials have contributed to its popularity, particularly among women.

*"Nowadays there are waterpipes especially for women; [they] are small, delicate and golden, with a more beautiful ornamented hose [as opposed to the waterpipe for men]. This is also a motivation [to smoke], maybe this form seduces the woman to smoke it, she might not like it, but it attracts her." *(Female smoker - urban area)

The ritual of decorating the waterpipe has become common which also made smoking much more appealing particularly to women who are lured by aesthetics.

*"... you put rose petals on the plate, you change its hose, paint it...At the end you'll like the way it looks. If the waterpipe looked nice and clean, then you'd have the desire to smoke it*." (Female smoker - rural area)

Innovations such as using cored pineapple and watermelon and shiny ornamentation and crystal bottles were "*encouraging use even if it does not taste better at the end, but psychologically [i.e. people feel that it tasted better just because in their view it looks better]" *(Male smoker - urban area).

Innovations in tobacco flavours also contributed to the increase in use and motivated initiation. The variety of available flavourings of waterpipe tobacco in the form of Ma'asal attracted smokers in trying them out and also enticed new smokers. More women than men smokers, and younger age category groups (men and women), as well as smokers more than non-smokers mentioned the variety of tobacco flavours of Ma'asal in the market as a reason for the increase in WTS. Ma'asal is fruit-flavoured, typically tobacco mixed with molasses. It gives off an odour of burned sugar when smoked. Ajami is the traditional unflavoured tobacco, with a harsher smell of tobacco. Ma'ssal tobacco is generally favoured by youth and women.

*"The companies are making use of it commercially, once they introduce the grape flavour to the market, people would then want to try it out, once they introduce the peach flavour, again people would want to try it out... I mean I think if they had only stopped with the apple flavour, maybe a lot of people would have had gave up waterpipe smoking...." *(Male non-smoker - urban area)

Contrasting the experience to that of smoking cigarettes, alluding to the different tastes of flavours, one waterpipe smoker said:

*"There is a difference in the taste [between a waterpipe and another] I have never through my entire life found a taste difference between one cigarette and another ... I mean the apple flavour is different than the melon, than the grape, than others... the waterpipe has a taste, and it is a good taste." *(Female smoker - urban area)

### Sensory qualities of waterpipe tobacco smoking

The sensory qualities of waterpipe smoking were important in encouraging use. The younger age groups and the smokers (regardless of age group) noted the sensory qualities evoked from waterpipe tobacco smoking as reasons that motivated smoking. Men and women equally attributed motivation to the sensory qualities such as taste, smell, sight of smoke, and bubbly sounds of the water in the bowl. Both smokers and non smokers equally cited taste and smell, however smokers more than non-smokers mentioned the sight and bubbly sounds.

*In the Shouf [Mountains in Lebanon], when we're sitting together in the evening, drinking something and it's quiet and tranquil... [pauses for a moment] it's sound is very nice... When you inhale it, and the water does this sound [referring to the sound of bubbling water]..." *(Male, non-smoker - urban area)

*".. I like the smoke when it comes out as it does... When I wasn't smoking the waterpipe, I used to look at someone who smoked it and see how the smoke comes out of it and that made me love it." *(Male smoker - rural area)

The taste and smell of Ma'asal tobacco were listed as main reasons for some to try the waterpipe, and eventually become addicted "*my parents used to sit and smoke the waterpipe ... Then from its nice smell we got hooked*" (Female smoker - rural area). The smell of the waterpipe even in public places was a motivator to initiate WTS:

*"When you arrive to a café, you smell the waterpipe from the outside, you say that's it, you want to smoke it" *(Male smoker - urban area)

### Media Influence

Men and women, smokers and non smokers, and the younger age groups noted the influence of the media - particularly advertisements, movies and TV series broadcast during the month of Ramadan - through its positive depictions of waterpipe tobacco smoking scenes. Much like the cigarette is portrayed as glamorous, sexy, and cool; the media also associated the waterpipe with glamour and lavishness "*What do the media do? [They make you] want to try, everything new they advertise... they tempt you with the design etc...*" The use of women in the media was common and a strategy used by media to motivate women to take up WTS *"... In an advertisement for any café, the first thing they show you is a girl smoking a waterpipe... The Media has a big influence..." *(Male smoker - rural area). Other brands were specifically developed and marketed for youth, for example like the new Ma'asal brand produced named "Shabablik "which means in Arabic "that which is youthful."

*"When it [Shabablik] was newly released into the market... the media has a big influence. And they seduce you, for example they give you a certain design for the package, they seduce you to try it, or maybe they do it in a cool attractive way ..." *(Male smoker - rural area)

The media also made the waterpipe more common and familiar to the people. In the early 1990's, during the month of Ramadan, scenes from cafés and restaurants serving the waterpipe were broadcast live across satellite TV in the middle east region which served to "advertise the waterpipe" and popularize it.

*"What really made the waterpipe in vogue over the last 10 years was the cafés, restaurants, Ramadan tents [coffee shops built temporarily during the holy month of Ramadan], these places were aired by TV stations, so... people were encouraged to go to such places. It [the waterpipe] has its own ambiance, they are promoting it."*(Male smoker - urban area)

### Policy

FGD participants noted that tobacco control policies should be set by the government and should regulate all forms of tobacco use, in particular that of waterpipe.

"it's the government's responsibility; the government should tackle such matters, raise awareness, take the necessary steps (Male non-smoker - rural area)

The commonly suggested policies that were brought up by participants were: banning WTS in closed indoor public places such as restaurants, raising price, limiting media influence be it direct or indirect advertising - most notably smokers in TV shows etc, as well as the need for health warning labelling like that required for cigarettes.

With regards to introduction of bans of smoking in public places, most participants viewed it as a measure that will decrease WTS.

*"...that would be the only thing that makes us--- if not quit it--- it would at least decrease by 50% or 60%..." *(Female smoker - urban area)

Also, the fact that both cigarettes and waterpipe were affordable in Lebanon was stated as one of the reasons why people continue to smoke. FGD participants stated that making WTS more expensive could potentially reduce use, although some were a bit sceptical since waterpipe smokers can prepare it at home as a cheaper alternative.

"I think if [the waterpipe] it is expensive relative to the income, it would not be that popular (...); a higher price would deter from use (Woman non-smoker - urban area)

FGD participants noted ubiquitous advertising of waterpipe tobacco particularly indirectly through propagation of positive images of WTS on TV programs.

"There are loads of advertisements for the Mo'assel tobacco, especially on the radio, more than TV (...). One would be tempted to try new flavors (Woman smoker - rural area)"

"Media and TV have a major impact. In any advertisement for a café they would show a woman smoking waterpipe. It is all about media (...). Media has a major impact on prevalence of waterpipe smoking, like when a new fashionable brand is out, This is the effect of media, you start trying out new trends., advertising has a major impact, they would entice you with a new design, so that you try it, ... (male smoker - rural area)

With regards to the need for HW's on waterpipe tobacco products, one participant compared that to presence of HW on cigarettes:

"Even the ministry of health, although they put a health warning on the cigarette packs, mentions nothing on waterpipe tobacco packs. .. although waterpipe smoking on a daily basis is more harmful than cigarette smoking (Woman non-smoker - urban area)"

Most importantly FGD participants stated that legislating policies and subsequent follow up and enforcement by the government to ensure compliance were essential to controlling WTS.

"[Tobacco control policies] have to be implemented. But such policies about banning waterpipe need to be enforced. There should be proper follow-up and enforcement by the government (male smoker - rural area)"

## Discussion

This manuscript has documented waterpipe smokers' and non smokers' perceptions about the rise in waterpipe tobacco smoking using qualitative methods. Qualitative methods are intended to show the variation in opinions, feelings, and experiences within a target population [18]. Despite not aiming for representativeness in the same sense as epidemiologic surveys, qualitative results are considered valid representations of a phenomenon of interest.

The results described herein have suggested specific themes that have influence non-smokers to start, and encouraged smokers to continue. There is a dearth of qualitative research exploring the meaning attributed to WTS and possible reasons for its spread in the Arab region and beyond. Quantitative research in Syria has specifically asked participants to describe why they think there was an increase in waterpipe smoking. They provided similar reasons as those of participants in this study such as availability, media, the social atmosphere, among others [21].

However, interestingly, although specific to waterpipe smoking, many of the themes described above are comparable to those manipulated by the cigarette industry to increase cigarette smoking, suggesting that the waterpipe industry has learned its lessons well from its predecessor. Finding from FGD's and interviews indicate that factors that are used to encourage WTS are similar to those that have been successful in enticing people to smoke cigarettes. For example, with regards to affordability, much research on the cost of cigarettes has indicated that "Price is one of the strongest influences on tobacco consumption (p.760)" [22]. This has led to pricing measures being included in the Framework Convention for Tobacco Control. Also our findings from focus group participants indicted that waterpipe ornamented hoses and other innovations in flavouring were designed to draw in consumers. Similarly, innovations in cigarette design have been key to the tobacco industry's success since the 19^th ^century [23] and have long been a strategy followed by multinational tobacco companies to encourage smoking. Research on tobacco industry documents has revealed that the industry has specifically altered cigarette design to target women [24], altering design features such as packaging, length, flavours, among others. Recently, flavoured cigarettes are an innovation of the tobacco industry [25]. More specifically, candy flavoured cigarettes have been manufactured to attract children [26]. With specific respect to packaging, the industry has made it more attractive "to appeal to women (p. i75)" [27]. Tobacco documents have similarly pointed to sensory characteristics as taste and smell as features manipulated by multinational tobacco companies to continue to attract consumers. For example, menthol cigarettes were introduced specifically for their sensory benefit [28].

Media promotion of waterpipe was stated by participants as attracting and enticing individuals to smoke. Much research on cigarettes has indicated the powerful force of advertising in promoting smoking. Pro-tobacco media has been shown to result in earlier initiation of smoking [29]. A cohort study of adolescents indicated that having a favourite cigarette advertisement in 1993 was positively related to smoking status in 1996 [30].

Understanding reasons behind the rise in waterpipe tobacco use is important if appropriate interventions are to be formulated. As evidence regarding the health effects of WTS increase, it becomes imperative that interventions are developed and tested to prevent initiation and promote cessation. Focus group participants referred to the lack of tobacco control policies to control waterpipe smoking as contributing to the waterpipe smoking epidemic. Evidence from cigarette use confirms their statements. Policy change has been documented to be the most effective prevention and control mechanism for tobacco use. Tobacco control policy should therefore be comprehensive and include waterpipe smoking. Banning WTS in public places and increasing taxation on waterpipe tobacco and price of waterpipes served in public places could stem the prevalence of WTS. Countries in Europe that have adopted smoking bans in public places have faced resistance in its application in waterpipe serving establishments [31] despite all evidence pointing to a need to incorporate bans of WTS in indoor public places [9].

Understanding what motivates people to smoke such as the sensory qualities described in our results will help shape anti-smoking messages. Any education campaign should also be coupled with market regulation to prevent the production of new flavours and innovations in design. Introduction of plain packaged waterpipe tobacco as well as waterpipe apparatuses with basic unornamented bodies could potentially reduce the allure of this method of smoking. Graphic health warnings on the waterpipe, the tobacco, and all accessories should also be required [32]. Findings from a study evaluating current practices in terms of health labelling on waterpipes and waterpipe products in Lebanon as well as few examples of waterpipe health warning practices in the UAE, South Africa, Canada, and Germany has revealed that health warning are too general, non-informative, very small in size, non-readable, and contain misleading labelling [32]. Such practices could indirectly lead users to believe that WTS is a safe alternative to cigarettes.

This research has implications for public health practice, advocacy, and research worldwide. A number of prevalence studies have indicated a rise in waterpipe smoking not only in the EM region but also in other developing and developed countries. For example, rising prevalence of waterpipe smoking among university students was reported by Jackson and Aveyard in the UK [5]. Findings in surveyed university students found that they had no knowledge regarding the health risks and were lured by its presence and availability within the student culture. In the US, prevalence studies are indicating up to 20% prevalence of waterpipe smoking in the last month among young adults [13]. In Syria, waterpipe smoking was regarded more as a pleasurable pass time with no regard for health consequences [14]. In addition, similar themes to those described herein were also observed in the qualitative findings of the larger project in other countries such as Syria, Egypt, and Palestine. Suggested specific themes that have come up from Lebanon that have influenced non smokers to start and encouraged smokers to continue are likely to be similar in other populations due to similarity of human's response to addiction. Worldwide people smoke cigarettes mainly for the same reasons: social norms and pressures that lead them to initiate while their young, curiosity to try leading to addiction; advertising and promotion; availability and accessibility; and lack of policy regulations.

There are several limitations to the research reported. One possible limitation is the inability to access the variation of lived experiences. However, focus groups or interviews were continued until saturation was achieved. Twenty-five focus groups is a large number in qualitative research and provide confidence in the breadth of experience achieved. Another main limitation is the potential bias from the researcher during analysis stages. However, this was minimized by having the entire research team construct the coding scheme. Social desirability is also a possible limitation as participants and interviewees may provide the response they believe the interviewer wants to hear. However, the interviewer had extensive experience in conducting qualitative focus groups and interviews and has been trained to listen without responding or giving cues, such as facial expressions or judgmental statements approving or disapproving participant views during data collection - thus limiting the expression of social desirability.

## Conclusions

This research documents waterpipe smokers and non-smokers explanations for the rise in WTS in Lebanon. They suggest motivations reminiscent of the lure of cigarette smoking, and driven by marketing of tobacco products. The increasing prevalence of WTS - particularly among youth, has been documented in countries of Europe and the US. Some of the same reasons stated in Lebanon for the rise of WTS could be playing out in other countries as well. Our results confirm the necessity of implementing the FCTC policies and strategies proposed by MPOWER across countries, and argue for strict inclusion of all tobacco products, including the waterpipe. National and international advocacy efforts are clearly needed to stem the waterpipe tobacco epidemic in Lebanon, the Arab region, and the world.

## Competing interests

The authors declare that they have no competing interests.

## Authors' contributions

RAA was the principal investigator on this research project. She was involved in conception, analysis, interpretation of data, and drafting and reviewing multiple drafts of this manuscript. RTN was involved in conception, analysis, and interpretation of data and was responsible for drafting the manuscript. JK did all the data collection, analysis and interpretation of data. She assisted in drafting and reviewing multiple parts of this manuscript. All authors read and approved the final version of the paper.

## Pre-publication history

The pre-publication history for this paper can be accessed here:

http://www.biomedcentral.com/1471-2458/11/315/prepub
